# “Exploring Nurses’ Perspectives on the Behavioral Responses of Residents with Intellectual Disabilities and Dementia in Social Welfare Centers”

**DOI:** 10.1192/j.eurpsy.2025.1550

**Published:** 2025-08-26

**Authors:** E. Naki, A. L. Batiridou, S. Mantzoukas, M. Gouva, Z. Konstanti

**Affiliations:** 1 Research Laboratory of Psychology of Patients, Families and Health Professionals, School of Health Sciences, Department of Nursing; 2 Research Laboratory of Integrated Health, Care and Well-being, School of Health Sciences, Department of Nursing, University of Ioannina, Ioannina, Greece

## Abstract

**Introduction:**

The behavioral disorders of individuals with dementia, as well as the attitudes and behaviors of people with intellectual disabilities, create challenges both in their home environment and for the nursing staff responsible for their care in Social Welfare Centers. This phenomenon is widely examined in both international and Greek literature; however, it remains a complex and multifactorial issue, not only in defining behavioral disorders but also in identifying the factors that cause them.

**Objectives:**

This qualitative study aims to explore how nurses at Social Welfare Centers perceive behavioral disorders, the factors they associate with them, and their emotional reactions. The goal is to gain an in-depth understanding of the phenomenon, highlight its significance, and bring forward the nurses’ perspectives.

**Methods:**

A qualitative approach was adopted to explore the relationship between nurses and beneficiaries due to behavioral disorders. A relational perceptual framework was used. Eight semi-structured interviews were conducted with permanent nursing staff from Social Welfare Centers. This was followed by a literature review of behavioral disorders, focusing on both nurses and beneficiaries.

**Results:**

The qualitative data analysis revealed three key themes: the role of the nurse and their relationship with beneficiaries in Social Welfare Centers, the behavioral disorders and emotional reactions of nurses based on their experiences, and the need for nursing staff education, along with the importance of an interdisciplinary team.

**Conclusions:**

The onset, worsening, reduction, or elimination of behavioral disorders depends on the complex interaction between nurses and patients with dementia or intellectual disabilities. The need to focus on factors that increase the occurrence of such behaviors is emphasized, to assist nurses working in long-term care facilities in managing them without compromising the quality of care provided to the beneficiaries. More studies are required to provide guidelines for better management of behavioral disorders.

**Disclosure of Interest:**

None Declared

